# Proteome analysis of the Gram-positive fish pathogen *Renibacterium salmoninarum* reveals putative role of membrane vesicles in virulence

**DOI:** 10.1038/s41598-022-06130-w

**Published:** 2022-02-22

**Authors:** Tobias Kroniger, Daniel Flender, Rabea Schlüter, Bernd Köllner, Anke Trautwein-Schult, Dörte Becher

**Affiliations:** 1 Institute of Microbiology, Department of Microbial Proteomics, Center for Functional Genomics of Microbes, University of Greifswald, 17489 Greifswald, Germany; 2 Imaging Center of the Department of Biology, University of Greifswald, 17489 Greifswald, Germany; 3 Institute of Immunology, Friedrich-Loeffler-Institute, Federal Research Institute for Animal Health, 17493 Greifswald - Isle of Riems, Germany

**Keywords:** Microbiology, Pathogens

## Abstract

Bacterial kidney disease (BKD) is a chronic bacterial disease affecting both wild and farmed salmonids. The causative agent for BKD is the Gram-positive fish pathogen *Renibacterium salmoninarum*. As treatment and prevention of BKD have proven to be difficult, it is important to know and identify the key bacterial proteins that interact with the host. We used subcellular fractionation to report semi-quantitative data for the cytosolic, membrane, extracellular, and membrane vesicle (MV) proteome of *R. salmoninarum*. These data can aid as a backbone for more targeted experiments regarding the development of new drugs for the treatment of BKD. Further analysis was focused on the MV proteome, where both major immunosuppressive proteins P57/Msa and P22 and proteins involved in bacterial adhesion were found in high abundance. Interestingly, the P22 protein was relatively enriched only in the extracellular and MV fraction, implicating that MVs may play a role in host–pathogen interaction. Compared to the other subcellular fractions, the MVs were also relatively enriched in lipoproteins and all four cell wall hydrolases belonging to the New Lipoprotein C/Protein of 60 kDa (NlpC/P60) family were detected, suggesting an involvement in the formation of the MVs.

## Introduction

*Renibacterium salmoninarum* is a 0.8–2 μm long, encapsulated, rod-shaped, non-motile Gram-positive fish pathogen that is the causative agent of bacterial kidney disease (BKD) in salmonids. Treatments for BKD have proven to be difficult as the pathogen is able to invade the host’s macrophages and survive and replicate intracellularly^1,2^. Further, the transmission of *R. salmoninarum* can occur vertically from parent to progeny via the egg^3^ and horizontally from fish to fish, e.g. via the fecal–oral route^4^. BKD has an impact on the sustainable production of salmonids for consumption^5^ where economic losses with up to 80% of *Oncorhynchus spp.* and 40% of *Salmo* *salar* stocks have been described^6^. While the processes during the pathogenicity of *R. salmoninarum* are not fully understood, several proteins are reported to be involved in the virulence of the bacterium. The immunogenic extracellular major soluble antigen of 57 kDa P57/Msa is widely considered as the dominant virulence factor of *R. salmoninarum* as the virulence of the bacterium correlates with the amount of the P57/Msa protein associated with the bacterial cell surface^7^ and also with the number of encoded gene copies of *msa*^8^. A second major virulence protein that is reported to be involved in immunosuppression of the host is a surface protein of 22 kDa named P22^9,10^. Both, P57/Msa and P22 are implicated in the suppression of the host antibody production and agglutination of leucocytes^9,11^. Other proteins that are involved in the virulence of *R. salmoninarum* include iron acquisition proteins^12^, hemolysins^13,14^, metalloproteases^14^, sortase and adhesion proteins^15^, capsular synthesis proteins^14^, catalase^2,14^, superoxide dismutase^14^, and thioredoxin peroxidases^14^.

For rational vaccine designs, it is crucial to identify the proteins that might be involved in host–pathogen interaction and to know their subcellular localization. For example, proteins located in the bacterial membrane (e.g. lipoproteins) or secretory proteins which directly interact with host cells are good targets for the development of vaccines^16,17^. While the translocation of bacterial proteins is typically mediated by the Sec or Tat secretion pathway or the more dedicated type I-V protein secretion systems^18^, proteins can also be secreted via bacterial membrane vesicles (MVs). Bacterial MVs are spherical extracellular bubbles known to be shed from Gram-negative and Gram-positive bacterial cell membranes and range in size between 20 and 400 nm^19^. While the mechanism behind the formation of MVs in Gram-positive bacteria is not fully understood, proteins involved in cell wall modification, like transpeptidases and autolysins are hypothesized to play an important role in the formation process, as these proteins have been detected in MVs released from Gram-positive bacteria^20–22^. Other contents of MVs include lipopolysaccharides^23^, peptidoglycan^24^, nucleic acids^24^, toxins^25^, and proteins^26,27^. Some of the proteins identified in MVs were shown to be relatively enriched within the MVs when compared to their parent bacterium, suggesting a selective packing of proteins into the MVs^26,27^. Similar to MVs of Gram-negative bacteria, MVs of Gram-positive bacteria and their cargo are reported to be involved in numerous processes^28^ including virulence^29^. The various contents of MVs and the presentation of putative immunogenic membrane proteins in a lipid environment^30^ make MVs a promising platform for vaccine development^21^.

Here, an extensive subcellular fraction of *R.* *salmoninarum* was performed to yield a comprehensive proteomic dataset which includes semi-quantitative data of the cytosolic, the membrane, and the extracellular subproteome as well as the subproteome of *R. salmoninarum* MVs. The global investigation of the most abundant proteins in different subcellular localizations may provide insight for vaccine or drug development research and give hints regarding the biogenesis of MVs in *R.* *salmoninarum* or in Gram-positive bacteria in general.

## Results

### Overall identifications and overlap between subcellular fractions

**Figure 1:**
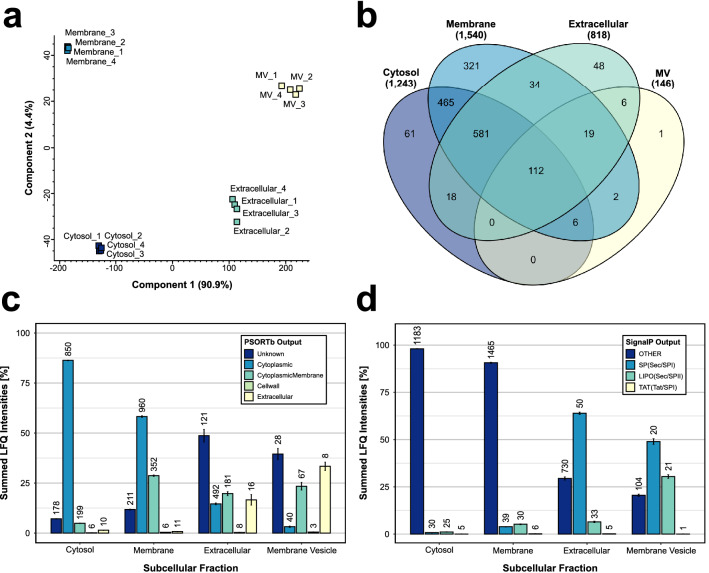
Overview of the protein identifications, predicted protein localizations, and predicted signal peptides for all enriched subcellular fractions: Protein abundances were only used for this figure when a protein was quantified in at least 3 out of 4 replicates in one or more of the four subcellular fractions. (**a**) Principal component analysis of the enriched subcellular fractions. Missing values were imputed from the normal distribution of each replicate (MV = membrane vesicle); (**b**) Overview of the overlap and the exclusively quantified proteins between the four enriched subcellular fractions (MV = membrane vesicle); (**c**) Illustration of the summed LFQ intensities of quantified proteins in the four subcellular fractions assigned to the respective predicted PSORTb localizations. The number of quantified proteins with the respective PSORTb prediction in each of the subcellular fractions is indicated above the bars; (**d**) Illustration of the summed LFQ intensities of quantified proteins in the four subcellular fractions assigned to the predicted SignalP protein signal peptides. The number of quantified proteins with the respective SignalP signal peptide prediction within the subcellular fractions is indicated above the bars.

This study is the first comprehensive proteome analysis of the *R. salmoninarum* strain ATCC 33209. By using subcellular fractionation, 1,781 of the 3,421 annotated proteins of *R. salmoninarum* were identified. Of these 1,781 identified proteins, 1,674 proteins were label-free quantified in at least 3 out of the 4 biological replicates in one or more of the four subcellular fractions, resulting in a quantification efficiency of 93.9%. A principal component analysis (PCA) of the label-free quantification (LFQ) intensities revealed a distinct differentiation between the four enriched subcellular fractions and a high reproducibility between the replicates within the respective fractions (Fig. 1a). Most proteins were quantified in the membrane fraction (1,540 proteins) followed by the cytosol (1,243), extracellular (818), and the MV fraction (146) (Fig. 1b). Several proteins were exclusively detected in one of the subcellular fractions. The highest number of exclusively quantified proteins were observed in the membrane fraction (321), followed by the cytosol (61), extracellular (48), and the MV fraction (1). To investigate an experimental enrichment of proteins and also to validate the success of the subcellular fractionation, the theoretical protein localizations were predicted *in silico* by using the tool PSORTb (version v3.0.2)^31^. For the annotated proteome of *R. salmoninarum*, the theoretical protein localizations were discriminated between “unknown” (781 total proteins), “cytoplasmic” (1,527), “cytoplasmic membrane” (1,035), “cell wall” (15), and “extracellular” (63) (Supplementary Table). A comparison between these theoretical protein localizations and the observed experimental protein localization in the enriched subcellular fraction demonstrated that proteins predicted to be cytosolic were enriched in quality and quantity in the cytosol and membrane fraction, but only some of these proteins were identified in a relatively low abundance within the extracellular and MV fraction (Fig. 1c). Further, in comparison to the cytosol fraction, a successful relative enrichment of membrane proteins was detected in the membrane fraction, as more membrane proteins were identified and these were relatively more abundant. The most abundant proteins in the extracellular and MV fraction were primarily predicted to be of unknown, membrane, or extracellular localization. Additionally, the presence of different secretion signal peptides was predicted by using the tool SignalP (version 5.0)^32^. According to the results of SignalP, 3,187 proteins in the proteome of *R. salmoninarum* are predicted to have no signal peptide (“Other”), 136 proteins are predicted to be Sec substrates cleaved by SPase I (“Sec/SPI”), 78 lipoproteins are Sec substrates cleaved by SPase II (“Lipo/SPII”) and 20 proteins are predicted to be Tat substrates cleaved by SPase I (“Tat/SPI”). These predictions were again matched with the findings of the mass spectrometry results. While the most abundant proteins in the cytosol and membrane fraction were without any signal peptide, proteins with a Sec/SPI and Lipo/SPII secretion signal were slightly enriched in quality and quantity in the membrane fraction compared to the cytosol fraction (Fig. 1d).

In the extracellular fraction, a clear enrichment of proteins with a Sec/SPI secretion signal was observed. Whereas only 6.1% (50/818) of the detected proteins were predicted to have this secretion signal, these proteins accounted for roughly 2/3 of the protein amount in this fraction. Proteins with a Sec/SPI secretion signal were also highly abundant in the MV fraction. Further, compared to the other subcellular fractions, similar amounts of proteins with Lipo/SPII secretion signal were identified in the MV fraction, but quantitatively a relative enrichment of these proteins was observed. Tables of the total identified proteins, the identifications in each of the subcellular fractions, the exclusive protein identifications in each of the subcellular fractions as well as the used PSORTb and SignalP predictions for the *R. salmoninarum* proteome can be found in Supplementary Table. The quantified proteins and the relative quantitative differences are also illustrated in a heat map in Supplementary Fig. 1.

### Subcellular enrichment of proteins involved in host–pathogen interaction

**Figure 2:**
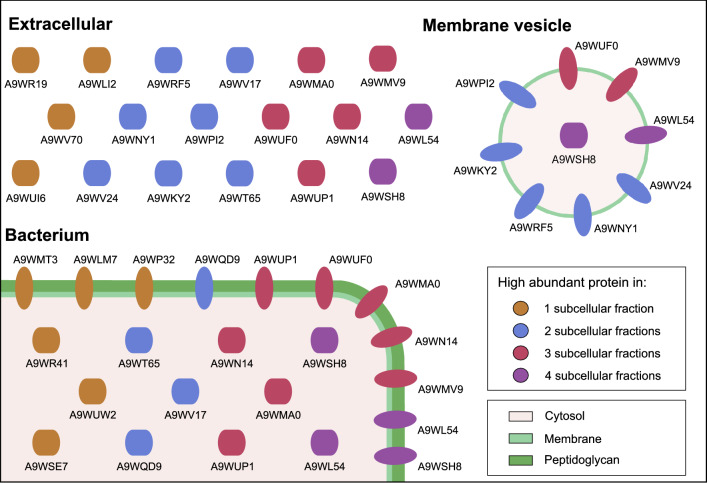
Schematic visualization of 25 high abundant proteins that are involved in virulence processes of *R. salmoninarum* and their experimental subcellular localization. Proteins are colored depending on their high abundance (higher than a log2 transformed median normalized LFQ intensity of 2) in one or more of the enriched subcellular fractions. Proteins are marked with their UniProt identifier.

For the understanding of the host–pathogen interaction, the analysis of secreted proteins and proteins associated with the surface of the bacterium is crucial. This analysis may further be important for new targeted approaches in drug development as inhibition of important proteins involved in host–pathogen interaction may lead to new treatment methods for BKD. The applied method of the subcellular fractionation provides information on the localization of proteins potentially involved in virulence and their relative abundance within the respective subcellular fraction. For a relative comparison of the protein amounts within the respective fractions, the LFQ intensities were median normalized within each of the subcellular fractions to take the enrichment effects during the sample preparation into account (Supplementary Table). The median normalization allows relative comparison between the subcellular fractions and can demonstrate enrichment effects of proteins in the fractions. Overall, 25 proteins that are associated with virulence processes were grouped and analyzed according to their high abundant occurrence in all, three, two, or only one of the enriched subcellular fractions (Fig. 2, Table 1).

**Table 1:**
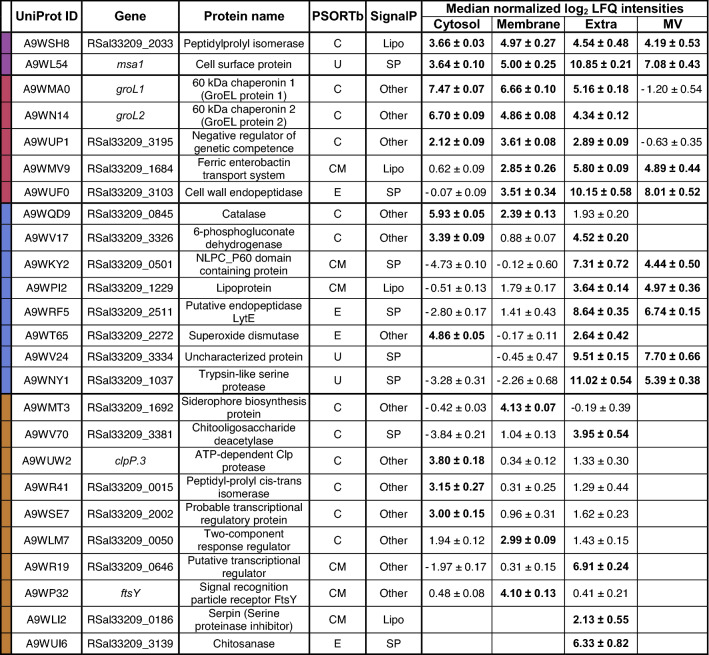
25 highly abundant proteins that are involved in virulence of *R.* *salmoninarum* and their subcellular localization

Proteins are with their predicted PSORTb localizations (U = unknown; C = cytoplasmic; CM = cytoplasmic membrane; E = extracellular), their predicted SignalP signal peptides (SP = secretory signal peptides transported by the Sec translocon and cleaved by Signal Peptidase I; Lipo = lipoprotein signal peptides transported by the Sec translocon and cleaved by Signal Peptidase II; OTHER = no detectable signal peptide) and the mean log2 transformed median normalized LFQ intensities in the four enriched subcellular fractions with their standard deviation between replicates (n = 4; Cyt = cytosol, Mem = membrane, Extra = extracellular, MV = membrane vesicles). Proteins are grouped and colored according to their abundance (higher than a log2 transformed median normalized LFQ intensity of 2, indicated in bold) in —4 of 4 subcellular fractions; —3 of 4 subcellular fractions; —2 of 4 subcellular fractions; ; —1 of 4 subcellular fractions. Within the groups, the listed proteins are sorted according to their theoretical subcellular localization.

The quantitative analysis of these 25 proteins within the subcellular fractions revealed that some proteins were only highly abundant in the cytosolic (UniProt ID A9WUW2; A9WR41; A9WSE7), membrane (A9WMT3; A9WLM7; A9WP32), or extracellular fraction (A9WV70; A9WR19; A9WLI2; A9WUI6). Other proteins were found in high abundance in both the cytosolic and membrane (A9WQD9), cytosolic and extracellular (A9WV17; A9WT65), or the extracellular and MV fraction (A9WKY2; A9WPI2; A9WRF5; A9WV24; A9WNY1). In addition, some proteins were detected in three of the subcellular fractions. The chaperonins 1 and 2 (A9WMA0; A9WN14) and a negative regulator of genetic competence (A9WUP1) were found in high abundance in the cytosolic, membrane, and extracellular fraction but not in the MVs. Additionally, a ferric enterobactin transport system (A9WMV9) and a cell wall endopeptidase (A9WUF0) were relatively enriched in the membrane, extracellular, and MV fraction, but not in the cytosol. Two of the 25 proteins have also been identified with high abundance in all four subcellular fractions. The most abundant of these two proteins is the cell surface protein P57/Msa (A9WL54), which is reported to be the major virulence factor of *R. salmoninarum*^7,11,33^. The other protein is an FK506 binding protein (FKBP) type peptidylprolyl isomerase (PPIase) (A9WSH8).

The mentioned results illustrate major differences in protein abundances between the enriched subcellular fractions. The major differences between the fractions emphasize the importance of proteins such as P57/Msa (A9WL54), which was identified in all subcellular fractions in high abundance, for the bacterium. Further, some proteins were enriched similarly in some subcellular fractions, but not in others. For example, while the chaperonin 2 (A9WN14) was abundant in the cytosol, membrane, and extracellular fraction, it was not detected in the MVs, indicating that this protein was neither actively sorted into the MVs nor it was present at the MV formation site. Further, correlations in relative protein enrichments between the extracellular and MV fractions can be observed, as many proteins like the immunosuppressive P22 protein were relatively enriched in the MVs and the extracellular fraction (Table 1). Nevertheless, other proteins like the chitin degrading proteins A9WV70 and A9WUI6 or a serpin (A9WLI2) were detected in high quantity in the extracellular fraction and were not detected in the MVs, demonstrating that the correlations of the MV proteome with the secretome are not an effect of the subcellular fractionation.

### Membrane vesicle proteome

**Figure 3:**
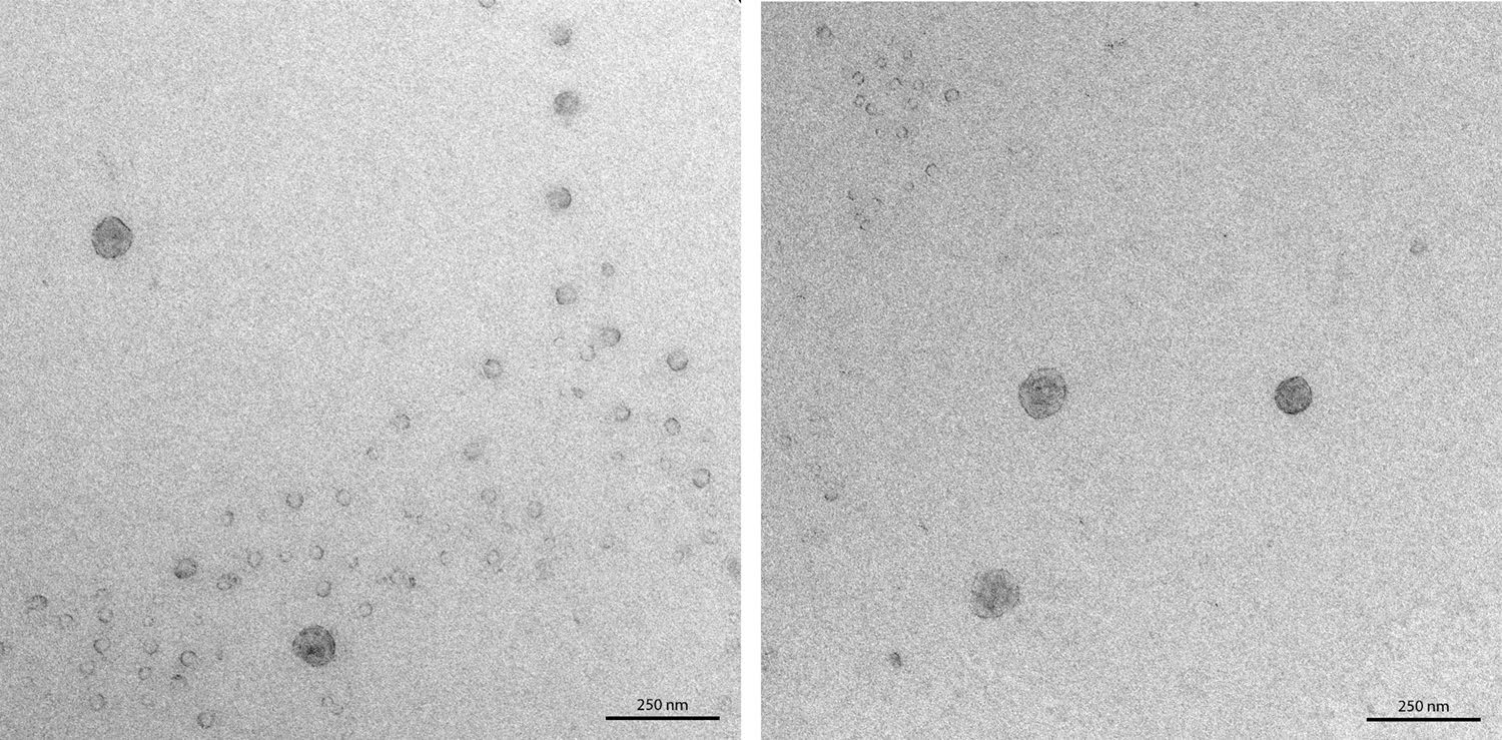
Transmission electron micrographs of isolated and purified membrane vesicles of *R. salmoninarum*.

The enriched and purified MVs of *R. salmoninarum* that were used for the MV proteome analysis ranged roughly in size between 20 and 100 nm (Fig. 3). In total, by using the described method to get distinct subcellular protein fractions, 146 proteins were found to be associated with the MVs. Remarkably, only 40 of these proteins were predicted to be cytosolic. The other proteins were mainly predicted to be membrane proteins (67 proteins) or of unknown localization (28) (Fig. 1c). Furthermore, the MVs were quantitatively enriched in lipoproteins compared to the other subcellular fractions (Fig. 1d) and 11 of the 30 most abundant proteins in the MVs were lipoproteins (Table 2).

**Table 2:** The 30 most abundant proteins quantified in the membrane vesicle (MV) fraction of *R. salmoninarum.*

Protein ID	Protein Name	PSORTb	SignalP	LFQ MEAN
A9WUF0	Cell wall endopeptidase	E	SP	26.85 ± 0.26
A9WV24	Uncharacterized protein	U	SP	26.54 ± 0.52
A9WUM2	Phosphate-binding protein PstS	E	LIPO	26.12 ± 0.26
A9WPE4	Oligopeptide-binding protein	U	OTHER	26.07 ± 0.28
A9WL54	Cell surface protein	U	SP	25.92 ± 0.16
A9WRF5	Putative endopeptidase LytE	E	SP	25.58 ± 0.23
A9WPC8	Glycine betaine-binding protein	U	LIPO	25.32 ± 0.56
A9WRF2	Uncharacterized protein	CM	LIPO	25.2 ± 0.38
A9WSK5	Putative iron(III) dicitrate-binding protein	CM	LIPO	25.01 ± 0.12
A9WLX2	Serine protease C	E	SP	24.4 ± 0.6
A9WNY1	Trypsin-like serine protease	U	SP	24.23 ± 0.2
A9WPI2	Lipoprotein	CM	LIPO	23.8 ± 0.14
A9WMV9	Ferric enterobactin transport system	CM	LIPO	23.72 ± 0.27
A9WM96	LytR_C domain-containing protein	CM	OTHER	23.42 ± 0.21
A9WKY2	NLPC_P60 domain-containing protein	CM	SP	23.27 ± 0.38
A9WSH8	Peptidylprolyl isomerase	C	LIPO	23.03 ± 0.25
A9WNV6	ABC transporter, permease protein	CM	OTHER	22.96 ± 0.32
A9WRT3	ABC transporter amino acid-binding protein	U	LIPO	22.7 ± 0.3
A9WP50	Signal peptidase I	CM	OTHER	22.44 ± 0.14
A9WR36	Hypothetical membrane protein	CM	OTHER	22.4 ± 0.52
A9WTJ9	Penicillin-insensitive transglycosylase	CM	OTHER	22.15 ± 0.42
A9WKU3	DsbA oxidoreductase	CW	LIPO	21.74 ± 0.56
A9WSE1	Protein-export membrane protein SecF	CM	OTHER	21.66 ± 0.42
A9WMK9	Cytochrome aa3 subunit 2	CM	LIPO	21.66 ± 0.48
A9WU60	Hypothetical exported protein	U	OTHER	21.65 ± 0.47
A9WLW9	Secretory peptidase	U	LIPO	21.56 ± 0.32
A9WSX9	DNA-directed RNA polymerase subunit beta'	C	OTHER	21.32 ± 0.28
A9WU66	Glycosyltransferase	CM	OTHER	21.24 ± 0.16
A9WTU3	Penicillin-binding protein	CM	SP	21.23 ± 0.38
A9WQS2	Transcriptional regulator, LytR family	CM	OTHER	21.23 ± 0.42

Proteins are listed with their UniProt-ID and annotated protein name, their predicted PSORTb localizations (U = unknown; C = cytoplasmic; CM = cytoplasmic membrane; CW = cell wall; E = extracellular), their signal peptides predicted by SignalP (SP = secretory signal peptides transported by the Sec translocon and cleaved by Signal Peptidase I; LIPO = lipoprotein signal peptides transported by the Sec translocon and cleaved by Signal Peptidase II; OTHER = no detectable signal peptide) and sorted by their mean log2 transformed LFQ intensities in the MV fractions (n = 4).

The most abundant protein found in the vesicles was the cell wall endopeptidase (A9WUF0), which belongs to the cell wall hydrolyzing New Lipoprotein C/Protein of 60 kDa (NlpC/P60) protein family (Table 2). Interestingly, the MV fraction was the only subcellular fraction in which all four NlpC/P60 proteins of *R. salmoninarum* (A9WUF0; A9WKY1; A9WKY2; A9WVI9) were identified. The second most abundant protein in the vesicles was the immunosuppressive P22 protein (A9WV24). The major virulence protein P57/Msa (A9WL54) was also in the top 5 most abundant proteins detected in the MVs. In addition, several transporters, proteases, and two proteins belonging to the LytR protein family were found high abundant within the MVs.

The MV proteome composition may give hints regarding the function and/or origin of the MVs, as proteins may actively be sorted by an unknown mechanism into MVs or that proteins are close to the site of MV biogenesis and are therefore detected in the MVs. Therefore, the MV proteome was examined for proteins that belong to the same or similar cellular functions of *R. salmoninarum*. For example, proteins involved in protein transport (A9WP49; A9WP50; A9WSE1; A9WSE2; A9WMN9; A9WST8), glycine betaine transport (A9WPC5; A9WQW0; A9WPC8), amino acid transport (A9WRT5; A9WRT3), oligopeptide transport (A9WPA0; A9WPE4), iron and hemin transport (A9WSK5; A9WMV9; A9WRJ4; A9WRJ5) and other ABC transporters (A9WUM2; A9WM30; A9WPI2; A9WPT7; A9WUY8; A9WNV6; A9WMU4; A9WPM2; A9WQR7) were found in the MVs. Additionally, proteins participating in FeS assembly (A9WT31; A9WT29; A9WT32), cell division (A9WRD6; A9WRE4; A9WTJ9) and other cell wall modifying processes (A9WRF5; A9WTU3; A9WTJ9; A9WLN3; A9WPT9; A9WUR8; A9WU60; A9WRE4; A9WM96; A9WQS2; A9WU75) were detected.

## Discussion

In this proteome analysis of *R. salmoninarum*, the subcellular localization of 1,674 proteins are reported with semi-quantitative data. The comparison of the composition and abundance of the detected proteins showed that proteins enriched within the subcellular fraction were also in silico predicted to be present. While the majority of proteins were detected in more than one subcellular fraction, the protein abundances between the subcellular fractions varied greatly in most cases. Interestingly, some proteins like the major virulence factor P57/Msa (A9WL54) and a PPIase (A9WSH8) were highly abundant in all analyzed subcellular fractions (Fig. 2), indicating an important role of these proteins for the bacterium. The role of PPIases in virulence is not fully understood. Some studies attribute a secondary role to these proteins, as they are involved in the folding and secretion of virulence factors as summarized by Unal and Steinert^34^. The FKBP type PPIase A9WSH8 has a high similarity to a FKBP type PPIase of the intracellular pathogen *Legionella pneumophila*. This PPIase of *L. pneumophila* is called macrophage infectivity potentiator (Mip) protein and is reported to potentiate the infection/replication within macrophages^35^. Also, a similar Mip protein was enriched in the MVs of *Neisseria meningitidis*^26^, is reported to be involved in the pathogenicity of the bacterium^36^ and lead to bactericidal activity in vaccination studies^37^. As *R. salmoninarum* can survive and replicate in macrophages^2^ and the A9WSH8 protein was found in all of the subcellular fractions in high abundance, the A9WSH8 protein may be a good target for further research.

Other findings, like the high abundance of the 60 kDa chaperonins 1 (A9WMA0) and 2 (A9WN14) in the cytosol, membrane, and extracellular fraction was obvious for an intracellular pathogen, as these proteins help to cope with the stressful environment within the host macrophages. Furthermore, two secretory chitin degrading proteins (A9WUI6; A9WV70) were found in abundance. Due to the large amounts of chitin and chitosan outside the gills and skin, it was already hypothesized that these proteins may be important for bacterial infection^38^. Moreover, the detected serpin (A9WLI2) plays a role in host–pathogen interaction as it is known to inhibit host proteases and interact with host serpins^39^. In addition, iron acquisition proteins are very important for pathogens, as the bioavailability of iron is stringently controlled by host proteins. The ferric enterobactin transporter (A9WMV9) and the putative iron(III) dicitrate-binding protein (A9WSK5) may play important roles in the iron acquisition of *R. salmoninarum* as they were found to be highly abundant in the membrane, extracellular, and MV fraction. Of the four described hemolysins of *R. salmoninarum*^14^, three hemolysins were detected (A9WUP1; A9WUL5; A9WQI5). While the neutral metalloproteinase (A9WUL5) was detected in the extracellular fraction and the hemolysin A (A9WQI5) in the cytosol and membrane fraction, the negative regulator of genetic competence (A9WUP1) was found in all subcellular fractions, indicating its significance for the bacterium.

A group of proteins known for the establishment of the infection of *R. salmoninarum* are the targets of the sortase A9WTU8, which covalently anchors the target proteins to the peptidoglycan surface. In the case of *R.* *salmoninarum*, sortase targets are implied to play a role in cell adhesion and colonization of the host. It was previously shown that the inhibition of the *R.* *salmoninarum* sortase (A9WTU8) results in a reduction of bacterial adhesion, invasion, and replication within chinook salmon embryo cells (CHSE-214)^15^. Three out of the eight putative sortase targets that are described for *R. salmoninarum* by Sudheesh *et al.*^15^ were identified in our study (A9WSP9; A9WUW6; A9WQT3), two of them were also identified in the MVs (A9WSP9; A9WUW6). Amongst these sortase targets, the putative peptidase A9WSP9 is of interest as it might be involved in the degradation of the major immunosuppressive protein P57/Msa. It has been described that P57/Msa is a substrate of an unknown putative autologous cell surface-associated serine proteinase which has a molecular mass of 100 kDa or greater and is cleaving the P57/Msa protein over time^40^. Interestingly, the putative peptidase A9WSP9 fits this description as it has an alpha/beta hydrolase fold domain, a mass of 101.37 kDa, and is anchored to the bacterial cell surface via an LPxTG cell wall anchor motif. In our data, A9WSP9 was detected with high abundance in all subcellular fractions. However, further experiments are necessary to prove this hypothesis.

The ability of *R.* *salmoninarum* to produce MVs has been reported recently^41^. Their study reported the packing of the major virulence factors P22 (A9WV24) and P57/Msa (A9WL54) into the MVs of *R. salmoninarum*^41^. Further, cytotoxic effects of the MVs on the salmon head kidney (SHK-1) cell line were shown^41^. The results presented here support the results of Echeverría-Bugueño *et al.*, as P22 and P57/Msa were also found in high abundance in the vesicles. While P57/Msa was highly abundant in all subcellular fractions, P22 was only high abundant in the MV and extracellular fraction, indicating that the MVs may have a role in virulence. The association of P57/Msa with the vesicles is noteworthy because it has been shown that the association with the membrane is crucial for the virulent properties of this protein^7^. However, while the study by Senson *et al.* shows the importance of the cell association of P57/Msa for the virulence, another study reported that nutritional mutant strains with cell-associated P57/Msa were avirulent, indicating that next to P57/Msa another yet uncharacterized factor needs to be present for virulence^42^. The protein that was only found within the MVs, the manganese-binding protein A9WVI9, belongs to the adhesin B protein family (InterPro family identifier: IPR006129). Proteins of this family are involved in bacterial adhesion and are enriched in other Gram-positive bacterial MVs^43^. Taken together, these findings support the current knowledge about the cytotoxic effects of the MVs on protein level and give hints for more targeted research regarding the putative involvement of the MVs during *R. salmoninarum* infection and development of BKD.

While the complete mechanism of MV shedding in Gram-positive bacteria is not fully understood, it is hypothesized that cell wall modifications (e.g. by transpeptidases and autolysins) play a critical role in the formation of MVs^20–22^. The aforementioned manganese-binding protein A9WVI9, which was the only protein exclusively found in the MVs, has an NlpC/P60 domain. In addition, more proteins of this major class of cell wall hydrolases were found to be highly abundant within the MVs (A9WUF0; A9WKY1; A9WKY2), indicating a role of proteins belonging to the NlpC/P60 family during MV biogenesis. Other cell wall modifying proteins found in the MVs were the penicillin-binding protein (A9WTU3), the penicillin insensitive transglycosylase (A9WTJ9), the hypothetical transglycosylase (A9WLN3), the peptidoglycan specific endopeptidase (A9WPT9), the D-alanyl-meso-diaminopimelate endopeptidase (A9WUR8), the hypothetical exported protein (A9WU60), the division-specific D,D-transpeptidase (A9WRE4), the FtsQ protein (A9WRD6), three proteins belonging to the LytR family (A9WM96; A9WQS2; A9WU75) and the putative endopeptidase LytE (A9WRF5). A study investigating the MV shedding in *Bacillus* *subtilis* reported that a LytE homolog, next to three other autolysins, was necessary for the MV production of the bacterium^22^. Together, these findings support the hypothesis that cell wall modifying proteins may be involved in the formation of *R. salmoninarum* MVs.

Taken together, we report the subcellular occurrence of 1,674 proteins of *R. salmoninarum* together with their relative abundance. This data can aid as a backbone for more targeted experiments aiming at the development of new drugs for the treatment of BKD or the understanding of the disease. The comparison of the 146 proteins identified from the MV fraction with other subcellular fractions showed that there is no continuous quantitative or qualitative correlation with any of them. Therefore, we can conclude that the composition of the MV proteome is unlikely randomly composed due to impurities. Further, we detected a relative enrichment of lipoproteins in the MV fraction. This may be especially important for a potential new route of vaccine development against BKD, as many bacterial lipoproteins are antigenic and immunoprotective^17^. In addition, vaccines based on liposomes or MVs are promising candidates because they cannot replicate and the proteins are presented in their native membrane environment^30^, which is important for their native structure^44^. Because of the comparatively simple production and enrichment of bacterial MVs, the application of MVs as a vaccine platform is discussed widely and shows promising results for other diseases and pathogens^45,46^. Until now, some attempts have been made to use MV vaccines in fish with varying results depending on the fish species^47–51^. One reason for the inconclusive data could be the variability of MV proteomes, as the protein composition has been reported to differ with growth phase^52,53^, cultivation media^54^, bacterial strain^55^, the MV extraction protocol^54,56^ or even the size of the vesicles^52^. Defined production conditions and detailed characterization of the MVs are therefore crucial for the successful application of MV vaccines. Still, the identification of P57/Msa protein in the MV fraction^41^ is an interesting result as a live vaccination approach with *R. salmoninarum* strains with normal and reduced cell surface presentation of P57/Msa showed the importance of the presence of P57/Msa for the protection during a challenge experiment in Atlantic salmon^42^. As both P57/Msa and P22 were detected in the MVs along with several adhesins and lipoproteins which are putatively immunogenic, the utilization of *R.* *salmoninarum* MVs as a new route for vaccination of fish against BKD is an interesting topic to examine in future in vivo studies.

## Methods

### Bacterial strain, cultivation, and harvest

For the bacterial (cytosol and membrane fraction) and the extracellular protein samples, the reference strain ATCC 33209 of *R. salmoninarum* was grown in quadruplicates in 100 ml PYC medium (1% peptone, 1% yeast extract, 0.1% L-cysteine HCl, pH 6.8)^57^ in a 15 °C water bath (Thermolab, GFL) under shaking at 150 rpm for ~ 145 h. For the preparation of the MV fraction, 450 ml per replicate (n = 4) was processed. Bacteria were harvested at an OD_600_ of 2.1 by centrifugation (Sorvall Lynx 6000, Thermo Fisher Scientific) at 10,000 × g for 20 min at 4 °C. The bacterial pellet was used to isolate the cytosolic and membrane protein subcellular fractions. The supernatant was filtered twice using a 0.45 µm bottle-top filter membrane (VWR) and used to prepare the extracellular and MV protein fractions.

### Preparation of cytosolic and membrane protein fractions

After resuspending the pellet in PBS, bacterial cells were disrupted using a Ribolyser (FastPrep-24 5G, MP Biomedicals) set to 6.5 m/s for 4 cycles at 30 s. In between the cycles, the suspension was cooled on ice for 5 min. After the last cycle, samples were centrifuged at 5,000 × g for 5 min at 5 °C to dispose the beads and cell debris. Afterward, the supernatant was ultracentrifuged (Sorvall Discovery M150SE, Hitachi) at 100,000 × g for 1 h at 4 °C. The supernatant contained the proteins referred to as “cytosolic fraction” in this study. The pellet was washed with HEPES (10 mM, pH 7.4) and ultracentrifuged again. The pellet contained the proteins referred to as “membrane fraction” in this study. Prepared fractions were stored at − 20 °C.

### Preparation of MV protein fraction

The filtered supernatant was concentrated ~ 20-fold using tangential flow filtration (Äkta flux, GE Healthcare) with a nominal molecular weight cut-off of 100 kDa (UFP-100-C-3X2MA, Cytiva) and ultracentrifuged afterward at 100,000 × g for 1 h and 4 °C. The pellet was washed in TE buffer (10 mM Tris–HCl, 1 mM EDTA, pH 7.25) and the supernatant was discarded. Subsequently, the MVs were purified by density gradient centrifugation with OptiPrep (60% Iodixanol). The MV containing pellet was resuspended in 2 ml 50% Iodixanol, diluted with a solution of 0.85% NaCl and 60 mM HEPES (pH 7.4). Then, carefully, 1 ml of 40%, 30%, and 10% Iodixanol diluted with 0.85% NaCl and 10 mM HEPES (pH 7.4) were carefully layered from the highest to the lowest Iodixanol concentration above the 50% solution layer. The samples were ultracentrifuged in a swing-out rotor at 100,000 × g for 2 h at 4 °C. The third and fourth 1 ml fractions contained the MVs and were transferred into new ultracentrifugation tubes, diluted five to sixfold with TE buffer, and ultracentrifuged again. Finally, the pellets of both fractions were pooled in TE buffer, transferred into a new reaction tube, and ultracentrifuged again. The MV containing pellet is referred to as “MV fraction” in this study and was stored at − 20 °C.

### S-Trap protein digestion and peptide fractionation

The S-Trap protein digest was performed according to manufacturer’s protocol (ProtiFi) with minor modifications. Briefly, 20 µg of protein of the cytosolic, membrane and MV fraction, determined by BCA assay according to manufacturer’s instructions (Thermo Fisher Scientific), was mixed 1:1 with 2 × lysis buffer (10% SDS, 100 mM TEAB, pH 7.55). Afterward, proteins were reduced in 20 mM DTT for 10 min at 95 °C and alkylated in 40 mM IAA for 30 min in the dark. Samples were acidified by the addition of phosphoric acid to a final concentration of 1.2% and diluted 1:7 with S-Trap binding buffer (90% methanol, 100 mM TEAB, pH 7.1). The proteins were digested with 1:50 trypsin in 50 mM TEAB for 3 h at 47 °C in S-Trap microcolumns and the peptides were eluted from the columns using 50 mM TEAB, followed by 0.1% aqueous acetic acid, and 60% acetonitrile containing 0.1% acetic acid. The peptides were dried using a vacuum centrifuge (Concentrator plus, Eppendorf).

To reduce the sample complexity, cytosolic, membrane, and MV samples were fractionated by performing basic reverse-phase peptide fractionation as described by the manufacturer (Pierce high pH reversed-phase peptide fractionation kit, Thermo Fisher Scientific). In short, peptides were loaded onto in-house packed C18 micro spin columns (Dr. Maisch HPLC GmbH ReproSil pur C18, pore size 300 Å, particle size 5.0 µm) and eluted in eight fractions with increasing acetonitrile concentrations ranging from 5 to 50% in a high-pH solution (0.1% triethylamine). The eluates of fractions 1 & 5, 2 & 6, 3 & 7 and 4 & 8 were pooled. Peptides were dried using a vacuum centrifuge, resuspended in 20 µl 0.1% acetic acid, and stored at − 20 °C until LC–MS/MS measurement.

### Preparation of the extracellular protein fraction and in-gel digestion

Extracellular proteins were enriched by using StrataClean affinity beads (Agilent) as described before^58^. In brief, 20 µl of primed StrataClean beads were incubated with 10 ml of filtered bacterial culture supernatant in an overhead shaker over-night at 4 °C. On the next day, the bead suspension was centrifuged for 45 min at 10,000 × g and 4 °C. Afterward, the pellet was dried using a vacuum centrifuge, and the proteins were separated by SDS-PAGE (Criterion, Bio Rad) with precast gels (Criterion TGX, Bio Rad). The separation was performed with 130 V until the solvent front reached the middle of the gel. The gel was fixated, Coomassie-stained, the lanes cut in five pieces of equal size, and tryptically digested as previously described^58^. The dried peptides were resuspended in 10 µl *Aq. dest.* and desalinated using C18 ZipTips according to the manufacturer’s protocol (Merck Millipore). Afterward, peptides were resuspended in 20 µl 0.1% acetic acid and stored at − 20 °C until LC–MS/MS measurement.

### Mass spectrometry data acquisition and analysis

Tryptic peptides of the cytosol, membrane, extracellular, and MV subcellular fractions were separated on an Easy nLC 1200 liquid chromatography system (Thermo Fisher Scientific) with a reverse-phase C18 column (in-house packed, inner diameter 100 µm, outer diameter 360 µm, length 200 mm, packed with Dr. Maisch ReproSil pur C18, pore size 120 Å, particle size 3.0 µm) and a column oven set to 45 °C. Peptides were loaded with 22 µl of buffer A (0.1% acetic acid) at 400 bar and subsequently eluted with a non-linear 100 min gradient from 1 to 99% buffer B (95% acetonitrile with 0.1% acetic acid) at a constant flow rate of 300 nl/min. Eluting peptides were measured in an Orbitrap Elite mass spectrometer (Thermo Fisher Scientific) in data-dependent mode. The MS1 scan was recorded in the orbitrap with a mass window of 300–1,700 m/z and a resolution of 60,000. The 20 most intense precursor ions (ions with an unassigned charge or a charge of 1 were excluded) were selected for CID fragmentation with a collision energy of 35%. The resulting MS/MS spectra were measured by the linear ion trap.

The resulting *.raw-files were searched against the UniProt reference proteome of the *R.* *salmoninarum* strain ATCC 33209 (ID UP000002007, 3,421 protein entries, download 17th August 2020) and the cRAP contaminants list using MaxQuant software (version 1.6.17.0)^59^. For samples of the cytosol, membrane and MV, the search was performed with a maximum of two missed cleavages, oxidation (M) and acetylation (protein N-term) as variable modifications and carbamidomethylation (C) as a fixed modification. The extracellular fraction was searched with a maximum of two missed cleavages, oxidation (M), acetylation (protein N-term), and carbamidomethylation (C) as a variable modification. Proteins were identified with a minimum of two peptides per protein group, with one unique peptide. Match between runs was enabled in between biological replicates. For protein quantification, unique and razor peptides were used and the label-free quantification (LFQ) calculation was performed separately for each of the enriched subcellular fractions.

The resulting data was analyzed with Perseus software (version 1.6.14.0)^60^. Data was filtered based on hits against the reverse database, identified by site and the contamination list of MaxQuant. Further, only proteins were treated as quantified if a protein group had LFQ intensities in at least 3 out of 4 replicates. LFQ intensities were log2 transformed and for relative comparison normalized by subtraction of the median.

### Transmission electron microscopy

For visualization of membrane vesicles, the method according to Thery *et al.*^61^ was applied with minor modifications. Briefly, isolated and purified membrane vesicles were fixed with 2% paraformaldehyde in 0.1 M sodium phosphate buffer (pH 7.5) and then allowed to adsorb onto a glow-discharged carbon-coated holey Pioloform film on a 400-mesh grid (Plano GmbH) for 20 min. The grid was then transferred onto two droplets of PBS, onto a droplet of 1% aqueous glutaraldehyde for 5 min, onto eight droplets of deionized water for 2 min each, and finally onto a drop of methyl cellulose-uranyl acetate (9 parts 2% methyl cellulose mixed with 1 part 4% aqueous glutaraldehyde) for 10 min on ice. After blotting with filter paper the grids were air-dried. All samples were examined with a transmission electron microscope LEO 906 (Carl Zeiss Microscopy GmbH) at an acceleration voltage of 80 kV. For acquisition of the images, a wide-angle dual-speed CCD camera Sharpeye (Tröndle) was used, operated by the ImageSP software. Afterward, all micrographs were edited using Adobe Photoshop CS6.

## Supplementary Information

Supplementary Information 1.

Supplementary Information 2.

## Data Availability

The mass spectrometry proteomics data have been deposited to the ProteomeXchange Consortium via the PRIDE^62^ partner repository with the dataset identifier PXD025586.
